# Staying Too Long – A Review of Delayed Discharges From Paediatric Wards

**DOI:** 10.1192/bjo.2024.503

**Published:** 2024-08-01

**Authors:** Harriette Pearson, Hilary Strachan

**Affiliations:** Manchester Foundation Trust, Manchester, United Kingdom

## Abstract

**Aims:**

Since 2020, there has been an increase in children with mental health presentations ending up on general paediatric wards. Hospitals are identified as a place of safety for young people in crisis, though admission to a paediatric ward is not without risk for the child and staff involved in their care. Stays are often prolonged and classed as delayed discharges. This evaluation looks at 22 admissions to general paediatric wards within an acute health trust in Greater Manchester.

**Methods:**

Local CAMHS teams identified 22 patients with a mental health presentation who had been admitted to paediatric wards and had delayed discharges between September 2021 and December 2023. Their electronic notes were analysed to identify number of bed days and CAMHS contacts, legal status, and discharge destination. Incident reports of each admission were analysed, and categorised into ‘Restraint/Rapid Tranquilisation’, ‘Assault on staff’ ‘Self harm’ ‘Abscondence’ and ‘Other’.

**Results:**

Of the 22 cases analyses, total bed days were 1469. The average number of bed days was 66.7. 6 admissions were over 100 days with the longest being 186 days. The majority (19) of the presenting complaints were categorised as ‘self-harm’ and /or ‘suicidal ideation’. The average number of core CAMHS contacts was 23 per admission, with an average of 9 consultant contacts, 5 Junior doctor out of hour contacts, and 32 meetings (e.g. discharge meeting, strategy meeting) requiring CAMHS attendance. 11 admissions involved assault on staff, with the highest number of assaults 48 during a single admission. 18 of the admissions required additional staffing (clinical support worker, security). Three patients required police to be called to the ward due to assault on staff. 9 of the patients were discharged to a social care placement, 8 were discharged home. The remaining were discharged to inpatient unit, day unit or to a family member.

**Conclusion:**

Mental health admissions to paediatric wards are associated with a high level of CAMHS contacts provided by Tier 3 staff, which creates a previously unseen burden on the service. Admissions can be prolonged. Patients are cared for in an environment which is not designed to meet their needs. This is demonstrated by high level of patients absconding from the ward and increased restrictive measures such as restraint and 1:1 observation. Admissions are also associated with high levels or assault on staff. Further work is needed to evaluate the economic impact of additional staffing on paediatric wards, as well as the impact on paediatric nursing and security staff.

